# Exploring Viral Metagenomics in Pediatric Patients with Acute Respiratory Infections: Unveiling Pathogens beyond SARS-CoV-2

**DOI:** 10.3390/microorganisms11112744

**Published:** 2023-11-10

**Authors:** Gabriel Montenegro de Campos, Debora Glenda Lima de La-Roque, Alex Ranieri Jerônimo Lima, Victória Simionatto Zucherato, Eneas de Carvalho, Loyze Paola Oliveira de Lima, Pedro de Queiroz Cattony Neto, Murilo Marconi dos Santos, Massimo Ciccozzi, Marta Giovanetti, Rodrigo Haddad, Luiz Carlos Júnior Alcantara, Maria Carolina Elias, Sandra Coccuzzo Sampaio, Dimas Tadeu Covas, Simone Kashima, Svetoslav Nanev Slavov

**Affiliations:** 1 Blood Center of Ribeirão Preto, Faculty of Medicine of Ribeirão Preto, University of São Paulo, Ribeirão Preto 14050-190, Brazil; gabrielmdecampos@usp.br (G.M.d.C.); debora.laroque@usp.br (D.G.L.d.L.-R.); vizucherato@gmail.com (V.S.Z.); skashima@hemocentro.fmrp.usp.br (S.K.); 2 Center for Scientific Development (CDC), Butantan Institute, São Paulo 05503-900, Brazil; alex.lima@butantan.gov.br (A.R.J.L.); eneas.carvalho@butantan.gov.br (E.d.C.); loyze.lima@butantan.gov.br (L.P.O.d.L.); pedro.cattony@butantan.gov.br (P.d.Q.C.N.); murilo.santos@butantan.gov.br (M.M.d.S.); carolina.eliassabbaga@butantan.gov.br (M.C.E.); sandracoccuzzo@butantan.gov.br (S.C.S.); dimas@fmrp.usp.br (D.T.C.); 3 Unit of Medical Statistics and Molecular Epidemiology, University Campus Bio-Medico of Rome, 00128 Rome, Italy; m.ciccozzi@unicampus.it; 4 Instututo Rene Rachou, Fundação Oswaldo Cruz, Belo Horizonte 30190-002, Brazil; giovanetti.marta@gmail.com (M.G.); alcantaraluiz42@gmail.com (L.C.J.A.); 5 Sciences and Technologies for Sustainable Development and One Health, Università Campus Bio-Medico di Roma, 00128 Rome, Italy; 6 Campus Ceilândia, University of Brasília, Federal District of Brazil, Brasília 70910-900, Brazil; haddad@unb.br

**Keywords:** viral metagenomics, acute infection, respiratory viruses, enteroviruses, respiratory syncytial virus, next-generation sequencing

## Abstract

The emergence of SARS-CoV-2 and the subsequent pandemic have prompted extensive diagnostic and clinical efforts to mitigate viral spread. However, these strategies have largely overlooked the presence of other respiratory viruses. Acute respiratory diseases in pediatric patients can be caused by a diverse range of viral agents, and metagenomics represents a powerful tool for their characterization. This study aimed to investigate the viral abundance in pediatric patients with acute respiratory symptoms who tested negative for SARS-CoV-2 during the Omicron pandemic wave. To achieve this, viral metagenomics and next-generation sequencing were employed on 96 nasopharyngeal swab samples, which were organized into 12 pools, with each pool consisting of eight individual samples. Metagenomic analysis revealed that the most prevalent viruses associated with acute disease in pediatric patients were respiratory syncytial virus (detected in all pools) and enteroviruses, which are known to cause significant morbidity and mortality in children. Additionally, clinically significant viruses such as mumps orthorubulavirus, human metapneumovirus, influenza A, and a wide array of human herpesviruses (1, 3–7) were identified. These findings highlight the extensive potential of viral metagenomics in identifying viruses other than SARS-CoV-2 that contribute to acute infections in children. Consequently, this methodology should garner clinical attention in terms of differential diagnosis and the development of public policies to address such conditions in the global pediatric population.

## 1. Introduction

The emergence of SARS-CoV-2 constituted a paramount public health emergency, necessitating the implementation of an unprecedented scale of viral testing and genotyping initiatives. In order to monitor the transmission of SARS-CoV-2 variants of concern, national molecular epidemiology programs were established, employing extensive viral sequencing methodologies. As a result, an astonishing number of SARS-CoV-2 complete genome sequences, surpassing 13 million, were generated and deposited in the GISAID EpiCoV database [1].

The prioritization of SARS-CoV-2 molecular testing and sequencing efforts has resulted in the neglect of testing for other significant respiratory viruses. Moreover, the observed decrease or absence of activity in expected viral waves, such as Respiratory Syncytial Virus (RSV) [2], can be attributed to the closure of schools during the pandemic lockdown. Previous studies have highlighted the co-circulation of multiple viruses during SARS-CoV-2 waves, particularly enteroviruses, rhinoviruses, and influenza A [3,4]. However, respiratory symptoms can be caused by specific viruses that may not be detectable using conventional respiratory virus panels. To identify clinically significant co-circulating respiratory viruses in nasopharyngeal samples that test negative for SARS-CoV-2, viral metagenomics represents the most suitable approach. This method utilizes unbiased amplification and provides a comprehensive overview of viral abundance in clinical samples without relying on molecular tests targeting specific pathogens [5].

In this study, we employed viral metagenomics to investigate the presence of co-circulating respiratory viruses among pediatric patients exhibiting acute respiratory symptoms, who tested negative for SARS-CoV-2. The research was conducted in the city of Ribeirão Preto, located in the state of São Paulo, Southeast Brazil, during the SARS-CoV-2 Omicron wave. Pediatric patients were selected as a suitable population to assess the prevalence of respiratory/enteric viral pathogens during the COVID-19 pandemic, given the heightened viral circulation and the clinical significance observed in this age group.

## 2. Materials and Methods

### 2.1. Clinical Sampling Criteria

In this study, we evaluated 96 nasopharyngeal swabs that were obtained from children with acute respiratory symptoms for the period of March 2022. The samples were collected by specialized professionals during routine SARS-CoV-2 detection involving standard procedure, which involved the use of swabs that were consequently inserted into viral transport medium (VTM) and transported to the diagnostic laboratory. All children were tested for SARS-CoV-2 RNA but showed a negative PCR result. This study was approved by the Institutional Ethics Commission of the Faculty of Medicine of Ribeirão Preto, University of São Paulo (process number CAAE: 50367721.7.1001.5440).

The inclusion criteria for the children were a negative SARS-CoV-2 test, mild to moderate clinical symptoms, and medical attendance only on ambulatory basis without hospital admission. The obtained nasopharyngeal swab was initially vortexed and submitted immediately for sample preparation of metagenomics.

### 2.2. Pre-Preparation of Nasopharyngeal Samples, Nucleic Acids Extraction, and NextSeq 2000 Illumina Sequencing

After vortexing, 600 μL of individual nasopharyngeal swab samples were pre-treated with Turbo DNase (Cat no AM1907, ThermoFisher Scientific, Waltham, MA, USA) for host/bacterial DNA removal. After DNAse inactivation, 8 individual nasopharyngeal samples were assembled into pools. The pooling of samples was performed randomly and was adopted in order to reduce the cost of the sequencing. Nucleic acids were extracted from the total pool volume using the High Pure Viral Nucleic Acid Large Volume Kit (Cat no 05114403001, Roche, Basel, Switzerland) in a final volume of 50 μL, applying minor modifications, i.e., the use of GenElute Linear Polyacrylamide carrier (LPA) (Cat no 56575, Merck, Rahway, NJ, USA) for nucleic acid concentration and isopropanol for precipitation. Five microliters of extracted nucleic acids were submitted to reverse transcription using the Superscript III First-Strand Synthesis System (Cat no 18080-051, ThermoFisher Scientific). The cDNA (extracted DNA) amplification was performed using the QuantiTect Whole Transcriptome Kit (Cat no 207045, QIAGEN, Hilden, Germany) applying a high yield isothermal amplification at 30 °C for 8 h. The sequence libraries were prepared using the DNA Prep Library Preparation Kit (20015829, Illumina, San Diego, CA, USA) and Nextera DNA CD Indexes (20015882, Illumina). The paired-end sequencing of the dual-indexed libraries was performed by Illumina NextSeq 2000 sequencing platform using the NextSeq P3 flowcell (300 cycles) (20038732, Illumina), following the manufacturer’s instructions.

### 2.3. Bioinformatic Pipeline for Taxonomic Classification of Viral Reads

The acquired raw sequence data underwent quality control inspection using FastQC v.0.11.8 [6] software. Subsequently, trimming and adapter removal were performed using Fastp v.0.20.0 [7] available at https://github.com/OpenGene/fastp (accessed on 12 April 2023). The following parameters were applied for Fastp: filtering all the reads with a phred quality score above 30, trimming of PolyX (3′) and PolyG tails and base correction [7]. The extraction of human reads (using NCBI id GRCh38.p14) was accomplished using BWA version 0.7.17-r1188 [8] available at https://github.com/lh3/bwa (accessed on 12 April 2023) and BWA-MEM [9] algorithm. The taxonomic classification of the unmapped reads (non-human reads) and the virome was inferred using Kraken2 v.2.0.8 [10] available at https://github.com/DerrickWood/kraken2 (accessed on 12 April 2023), employing the genomic NCBI RefSeq database (last updated April 2022). Additionally, Kraken2 was employed to subtract any remains of human, bacterial, and parasitic reads, which were subsequently excluded from further analysis. Non-human infectious viruses, phages, and artifacts were manually eliminated, resulting in a curated virome limited to human viruses.

Finally, for viral abundance analysis, bar plots were generated using R programming language version 4.2.2—“Innocent and Trusting” [11], along with RStudio [12]. The following libraries were utilized: readr [13], tidyverse [14], and dplyr [15] for handling, editing, and creating datasets. Bar plots were created using ggplot2 [16], while ggsci [17] was employed for color selection.

## 3. Results

### 3.1. Sociodemographic Characteristics of the Tested Patients

The pediatric patients resided in the region of Ribeirão Preto city, located in the state of São Paulo, Southeast Brazil. The average age of the children was 5 years old, with a range of 2 months to 12 years. The gender distribution among the participants was 54.2% boys and 45.8% girls.

### 3.2. Bioinformatic Analysis

The NGS performed on the pools yielded an average of approximately 2.1 million total reads, which proved sufficient for metagenomic investigations. The quantitative properties of the NGS data, following read trimming, extraction of non-viral reads, and the obtained viral reads, as reported by Kraken2, are summarized in Table 1. Across all pools, the median proportion of viral reads accounted for 0.495% of the total of raw read count. Notably, pools 2, 8, 9, and 10 exhibited a higher abundance of raw reads; however, pools 1, 3, 6, and 11 exhibited a higher abundance of viral reads.

### 3.3. Viral Abundance

**Figure 1:**
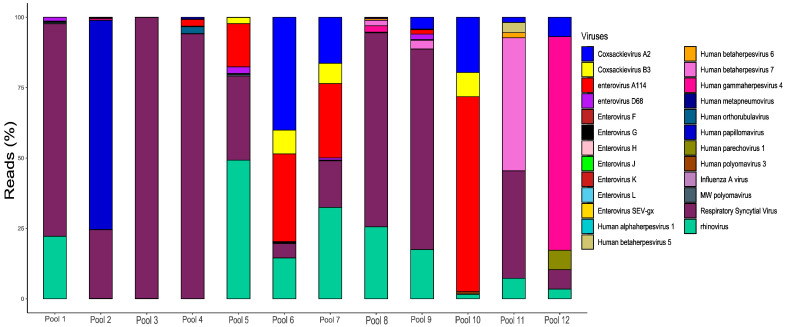
Bar plot of the abundance of the curated virome of clinically important viruses that were identified among acutely infected pediatric patients negative for SARS-CoV-2. The universal presence of respiratory syncytial virus among all pools can be observed. Human rhinoviruses (A1, B3 and C1) and human papillomaviruses (types 5, 9, 16, 48, 50, 126, 137, 172, and 178) were united in rhinovirus (Caribbean blue) and human papillomavirus (Medim blue).

To gain a comprehensive understanding of clinically significant viruses present in pediatric patients experiencing acute respiratory symptoms, we conducted a meticulous curation of the overall virome, excluding any artifacts or contaminants. Figure 1 illustrates the curated virome, specifically focusing on viruses capable of causing human infections, within the cohort of patients displaying acute respiratory symptoms.

**Figure 2:**
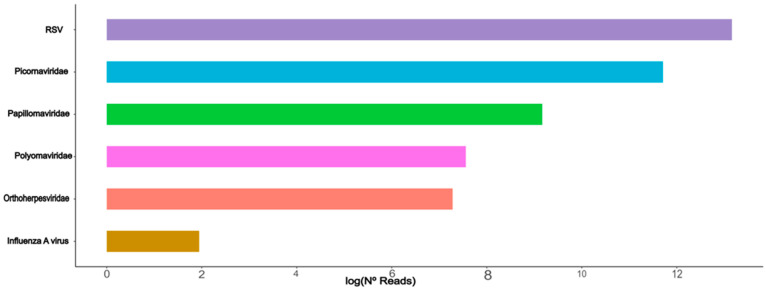
Horizontal bar plot of the most abundant curated clinically important viruses that were identified among acutely infected pediatric patients negative for SARS-CoV-2. The expressive presence of respiratory syncytial virus can be observed (*X*-axis: Natural log of the number of obtained trimmed reads).

Among the clinically significant viruses, the RSV and picornaviruses (specifically rhinoviruses and enteroviruses) exhibited the highest levels of abundance across all pools (Figure 2). Notably, the enterovirus group encompassed several identified viruses, including coxsackievirus A2, B3, enterovirus A114, D68, F, G, H, J–L, and SEV-gx. The median percentage of reads assigned to these enteroviruses across all pools was 4.6% (of all pools), with pools 7 and 10 displaying the highest percentages at 79.89% and 97.5%, respectively. As for RSV, the median percentage of reads allocated to this virus was 17.5% among all tested pools. Remarkably, pools 3 and 4 exhibited nearly exclusive identification of RSV, accounting for 99.88% and 94.08% of reads, respectively (Figure 1). The third most prominent group was the picornaviruses, which included human rhinovirus types A (1, 2), B (3), C (1), and D. These picornaviruses collectively represented a median percentage of 14% of the reads.

At lower frequencies (ranges between 0.02% and 8.5%), we successfully identified several clinically significant viruses that impact pediatric patients. In pools 2 and 4, we detected Human orthorubulavirus types 2 and 4, known as causative agents of mumps. Additionally, we identified Human metapneumovirus (pool 1) and influenza A (pool 2), both of which are responsible for acute respiratory infections in children. Our analysis also encompassed a broad spectrum of herpesviruses, including Human herpes virus 1 (HHV-1), Human herpes virus 3 or varicella-zoster virus (HHV-3), Epstein–Barr virus (Human herpesvirus 4, HHV-4 or EBV), cytomegalovirus or Human herpesvirus 5 (HHV-5 or CMV), and Human herpesviruses 6 and 7. Furthermore, in pools 1 and 5, we successfully detected the MW Polyomavirus (MWPyV), while Polyomavirus type 3 was identified in pool 10. Additionally, seven pools (58.3%) exhibited the presence of papillomaviruses, notably papillomavirus type 5 (HPV-5), indicating the possible presence of genomic material originating during specimen collection. We were able to identify Human Parechovirus 1 (HPeV-1) only in pool 12.

**Figure 3:**
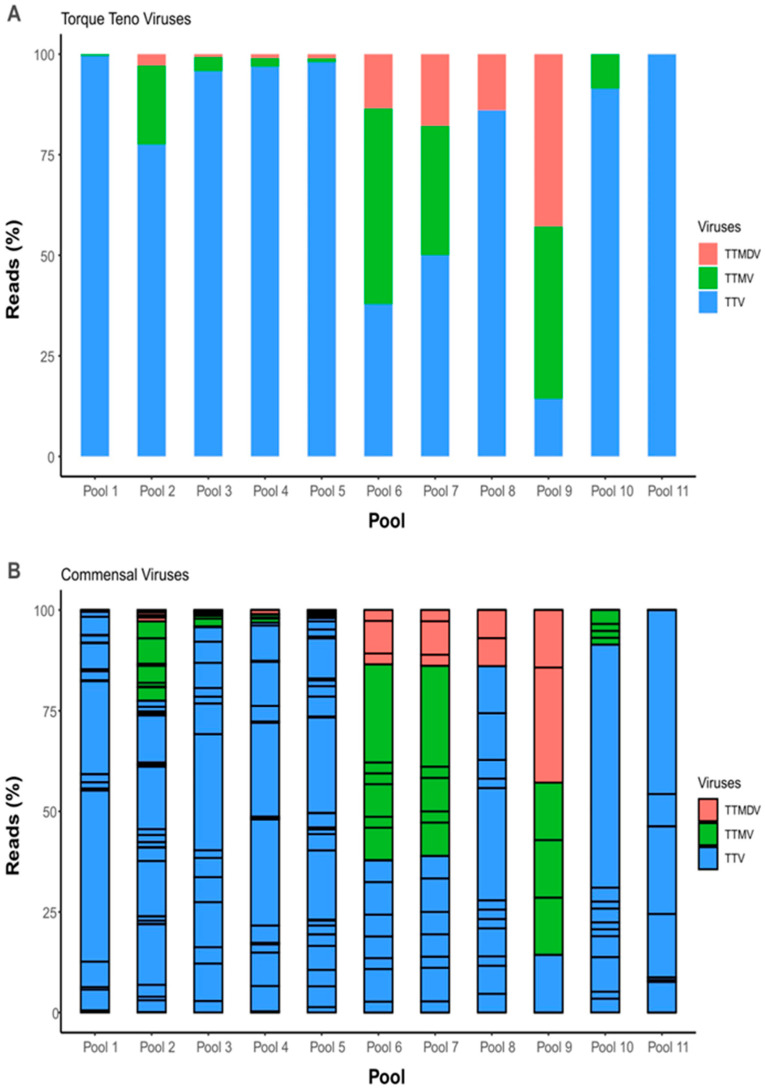
Bar plot of the abundance of commensal viruses (anelloviruses) detected in acutely infected children negative for SARS-CoV-2. (**A**) At genus level (Alpha, Beta, and Delta torquetenovirus). (**B**) At species level.

We also assessed the predominant commensal viruses, which were detected in all pools except for pool 12 (absence of commensal viral reads). Among the torque teno viruses (TTV), the most prevalent genogroups were TTV-1–25 and 27–29. This was followed by torque teno midi viruses (TTMDV) (1, 2, 4, 6–15) and torque teno mini viruses (TTMV) (1–12, 16, 18, and 27). The relative abundance of these commensal viruses in nasopharyngeal swabs collected from pediatric patients is presented in Figure 3A,B. For more information of the different species identified among each genus, observe Supplementary Table S1.

## 4. Discussion

In this study, conducted during the Omicron wave in Southeast Brazil, we employed viral metagenomics to identify clinically important viral agents in pediatric patients who tested negative for SARS-CoV-2. Through this approach, we successfully identified a diverse range of viruses, highlighting the utility of metagenomics for viral discovery. This not only should redirect the clinical focus of pediatricians away from solely SARS-CoV-2, but also aids in elucidating the complete clinical picture in affected patients.

A high diversity of anelloviruses, particularly TTV, was identified with relatively fewer reads for TTMV and TTMDV. Anelloviruses are generally considered non-pathogenic commensal viruses, and their detection rates can reach as high as 90% in the saliva of healthy children [18]. Our findings suggest that anellovirus infection is likely acquired early in childhood, possibly through close daily contact, as proposed by previous studies [19,20]. On the other hand, TTV has frequently been detected in pediatric patients with respiratory infections [21] and fever [22]. Interestingly, TTV has been found in similar read abundance among both healthy children and pediatric patients with pneumonia and asthma, indicating a commensal role rather than a pathogenic effect for this virus [23].

Among the clinically significant viruses, we have identified two viral families that exhibit a prominent concentration of sequences in the pediatric patients we tested. These families are *Picornaviridae* (specifically the *Enterovirus* genus, including enteroviruses A, B, D, and rhinoviruses A–C) and *Pneumoviridae* (human orthopneumovirus, or RSV). These viruses are considered crucial pathogens in acute respiratory infections affecting children. Our analysis revealed a high abundance of reads belonging to RSV across all sample pools. RSV is recognized as the primary cause of acute respiratory disease in children, leading to significant morbidity [24,25]. Its impact is particularly severe in pediatric patients with oncologic diseases, often resulting in fatal outcomes [26]. Our analysis confirmed the high frequency and importance of RSV, as it was discovered at a significant rate in all tested sample pools.

The detected enteroviruses displayed considerable diversity, primarily represented by coxsackievirus A2, B3, enterovirus A114, and D68. These viruses are highly transmissible during early childhood, causing significant morbidity and large outbreaks among children [27,28,29,30,31]. Enteroviruses exhibit a seasonal pattern, predominantly causing infections in the summer and autumn in temperate regions, and persisting throughout the year in tropical zones [32]. Outbreaks of enterovirus infections have been documented in infants and young children in Taiwan (2004–2012; 2018), the United States of America (2014–2015; 2015–2016), and Spain (2004–2014). Vertical transmission from infected newborns is the usual route of these outbreaks [32]. Enterovirus A114, a strain recently identified in 2016, remains relatively understudied, and there is limited information available about its clinical symptoms [33]. Enterovirus infections can cause a wide range of acute clinical conditions, with some individuals remaining asymptomatic. The most prominent manifestation is a severe respiratory infection, which can occasionally progress to neurological complications (such as flaccid paralysis) or cardiovascular complications (such as myocarditis). Other clinical manifestations may include fever or hypothermia, irritability, lethargy, anorexia, rash, jaundice, apnea, hepatomegaly, abdominal distension, emesis, diarrhea, decreased perfusion, and hand–foot–mouth disease [32,34,35,36].

In addition to the previously mentioned viruses, we detected a considerable number of viruses that are significant human pathogens and can cause acute infections in pediatric patients. These include mumps orthorubulavirus, human metapneumovirus, and various human herpesviruses, specifically types 1, 3–7. The mumps orthorubulavirus identified in this metagenomic study is the cause of an acute vaccine-preventable childhood viral disease, i.e., mumps, characterized by painful swelling of the parotid glands [37]. Among the identified herpesviruses, all of them are transmitted through oral and/or respiratory routes, primarily during childhood via contaminated droplets [38]. These infections can lead to a wide range of symptoms [38]. Additionally, we found sequences of the MW polyomavirus, which has an unknown clinical impact in pediatric patients, but it is presumed to occur during early childhood [39,40], consistent with our findings. Although we identified Human papillomavirus (HPV) in our samples, the specific types detected are not oncogenic [41] They belong to the low-risk HPV category, typically causing benign lesions in children [42]. As well as Enteroviruses, the detected Human parechovirus-1 is also a representative of the *Picornaviridae* family, transmitted by fecal–oral and possibly by oral–oral (respiratory) routes [43]; it is known for causing a spectrum of disease in humans, including general respiratory illness in neonates and young infants [43,44].

An important observation of our study was that we did not analyze possible bacterial infections that might also cause acute respiratory illness in pediatric patients. One of the reasons is that we did not optimize a pipeline designated to classify Monera Kingdom reads, and it was not part of the original scope of our study. However, we were able to analyze the taxonomic classification of bacterial reads and identify possible bacterial pathogens that can cause acute disease in children, the most important of which were *Streptococcus pneumoniae*, *Legionella pneumophila*, *Moraxella catarrhalis*, *Klebsiella pneumoniae*, and *Haemophilus influenzae*, among others [45]. This shows that bacterial pathology is also of significant importance for acute respiratory disease in children, and future investigations are needed to depict the bacterial landscape among acutely infected pediatric patients.

A limitation of our study was that the analyzed samples were pooled in order to reduce the cost and to test simultaneously more clinical specimens thus precluding individual identification of the viral pathogens and a consequent link to the respective clinical condition. On one hand, the idea of this study was to show the overall viral abundance with the most represented respiratory viruses with pediatric involvement beyond SARS-CoV-2, and a causal link between identified viral reads and clinical symptoms was not considered during the study design. On the other hand, we identified only viral genetic material along with the presence of probable bacterial coinfections that additionally hampers the establishment of a relationship between the identified virus and the clinical symptoms. Nevertheless, the obtained results, especially for the most represented viruses might be useful for pediatricians in determination of the correct or differential diagnosis during clinical examination of affected children with the establishment of adequate diagnostic tests, interventions, and therapeutic regimens.

## 5. Conclusions

In conclusion, our findings highlight the continued importance of studying and identifying co-circulating respiratory viruses, despite the global awareness of the SARS-CoV-2 pandemic. Our study revealed the presence of several other viruses that cause acute infections in pediatric patients. Among these, RSV and a diverse array of enteroviruses were the most abundant. These findings emphasize the significance of considering these enteroviruses as important pediatric pathogens in the differential diagnosis of acute infections. Furthermore, our study identified other clinically significant viruses, including herpesviruses and mumps, which can cause acute infections in children. This underscores the value of metagenomic approaches in identifying novel, unexpected, or re-emerging viruses in pediatric patients with acute respiratory diseases, which could be used in a viral surveillance scenario. Such comprehensive analyses contribute to our understanding of the viral landscape and aid in better diagnosing and managing acute respiratory diseases in the pediatric population.

## Figures and Tables

**Table 1:** Quantity of viral and bacterial reads obtained before and after the quality control and mapping of the tested pools.

Pool Number	Number of Raw Reads	Number of Reads after Filtering and Trimming	Unmapped Reads	Bacterial Reads	Viral Reads *
1	141,414,844	50,264,820	5,361,942	2,348,074	426,058 (7.95%)
2	173,501,138	50,794,796	12,795,706	7,737,515	107,842 (0.84%)
3	110,099,280	41,203,494	1,171,004	314,263	230,229 (19.66%)
4	155,965,396	57,456,570	1,276,254	233,930	13,540 (1.06%)
5	157,121,534	45,602,648	11,980,966	11,377,822	46,637 (0.39%)
6	133,245,536	52,254,492	1,792,958	726,428	67,971 (3.79%)
7	152,975,136	55,806,062	1,087,668	125,481	4312 (0.4%)
8	173,144,838	67,286,008	1,570,629	308,187	3522 (0.22%)
9	182,336,750	70,868,210	1,651,346	287,555	1447 (0.09%)
10	169,962,646	61,872,152	1,214,222	244,324	5148 (0.42%)
11	147,440,478	51,462,644	1,410,016	571,614	21,120 (1.5%)
12	153,340,198	60,556,652	24,716,749	6,591,749	107,425 (0.43%)

* Percentage calculated as referred to the unmapped reads.

## Data Availability

Not applicable.

## Supplementary Materials

The following supporting information can be downloaded at: https://www.mdpi.com/article/10.3390/microorganisms11112744/s1, Table S1: Anellovirus abundance among pools.
